# Three-Dimensional Kinematics of the Human Metatarsophalangeal Joint during Level Walking

**DOI:** 10.3389/fbioe.2014.00073

**Published:** 2014-12-15

**Authors:** Sivangi Raychoudhury, Dan Hu, Lei Ren

**Affiliations:** ^1^School of Mechanical, Aerospace and Civil Engineering, University of Manchester, Manchester, UK

**Keywords:** metatarsophalangeal joint, three-dimensional kinematics, position and orientation, walking, ground reaction force

## Abstract

The objective of this study is to investigate the three-dimensional (3D) kinematics of the functional rotation axis of the human metatarsophalangeal (MP) joint during level walking at different speeds. A 12 camera motion analysis system was used to capture the 3D motion of the foot segments and a six force plate array was employed to record the simultaneous ground reaction forces and moments. The 3D orientation and position of the functional axis (FA) of the MP joint were determined based on the relative motion data between the tarsometatarsi (hindfoot) and phalanges (forefoot) segments. From the results of a series of statistical analyses, it was found that the FA remains anterior to the anatomical axis (AA), defined as a line connecting the first and fifth metatarsal heads, with an average distance about 16% of the foot length across all walking speeds, and is also superior to the AA with an average distance about 2% of the foot length during normal and fast walking, whereas the FA shows a higher obliquity than the AA with an anteriorly more medial and superior orientation. This suggests that using the AA to represent the MP joint may result in overestimated MP joint moment and power and also underestimated muscle moment arms for MP extensor muscles. It was also found that walking speed has statistically significant effect on the position of the FA though the FA orientation remains unchanged with varying speed. The FA moves forwards and upwards toward a more anterior and more superior position with increased speed. This axis shift may help to increase the effective mechanical advantage of MP extensor muscles, maximize the locomotor efficiency, and also reduce the risk of injury. Those results may further our understanding of the contribution of the intrinsic foot structure to the propulsive function of the foot during locomotion at different speeds.

## Introduction

The human foot is an enormously complex structure consisting of numerous bones, muscles, ligaments, and synovial joints. As the only body component in contact with the ground, it plays multiple crucial roles in attenuating ground impacts, maintaining locomotor stability, and generating propulsive powers during locomotion (Ker et al., 1987; Carrier et al., 1994; Ren et al., 2008a). Over the past decades, many experimental and computer simulation studies have been conducted to investigate the locomotor function of the human foot complex (Apkarian et al., 1989; Scott and Winter, 1993; Leardini et al., 1999; Gefen et al., 2000; Carson et al., 2001; MacWilliams et al., 2003; Nester et al., 2007; Ren et al., 2010; Qian et al., 2013). However, most of those studies have mainly concentrated on the biomechanics of the whole foot segment, whereas the specific functioning of the distal part of the foot has been much less frequently studied.

The metatarsophalangeal (MP) joint near the distal end of the foot may have multiple functions during locomotion (Bojsen-Møller and Lamoreux, 1979; Mann and Hagy, 1979; Stefanyshyn and Nigg, 1997). During walking, the MP joint undergoes progressive dorsiflexion in the stance phase, which tightens up the plantar aponeurosis to wrap around the metatarsal heads. This may help to elevate and stabilize the longitudinal arch of the foot by using the windlass mechanism of the plantar aponeurosis without muscle function (Mann and Hagy, 1979). The dorsiflexion of the MP joint in the late stance phase can noticeably reduce the moment arm of the ground reaction force compared to a single rigid foot lever. This may increase the effective mechanical advantage (EMA) of ankle dosriflexor muscles and hence reduce the muscular effort during push-off (Bojsen-Møller and Lamoreux, 1979; Biewener, 1989). In addition, it was found that the dorsiflexion of the MP joint tightens the connective tissue framework around the ball of the foot, and thereby constraints the relative motions of the skin to enable shear forces to be transmitted to the skeleton (Bojsen-Møller and Lamoreux, 1979).

The MP joint may play significant roles during rapid change of body movement because the toes help in balancing the body while the body is changing its motion rapidly (Mann and Hagy, 1979). It was found that the MP joint is a significant absorber of energy in sprinting, with increased energy absorption with increased speed (Stefanyshyn and Nigg, 1997). Furthermore, it was suggested that ankle and MP joints may provide a mechanism of varying the gear ratio of the ankle dorsiflexor muscles during running (Carrier et al., 1994). This may enhance locomotor performance during constant speed running by maintaining the muscles near the high-efficiency portion of the force–velocity curve.

To simplify the analyses, many of the previous studies have assumed that the MP joint rotates about an axis perpendicular to the sagittal plane, originating from the fifth metatarsal head (Stefanyshyn and Nigg, 1997, 1998). This two-dimensional assumption simplifies the motion of the MP joint and does not reflect the oblique nature of the MP joint. A recent study investigated the effect of the MP joint axis by comparing the calculated joint kinetic variables based on different MP joint axis definitions in sprinting (Smith et al., 2012). It was found that MP joint axis has a significant effect on calculated joint moment, power, and energy, and oblique joint axes result in less energy absorbed at the MP joint than a simplified perpendicular axis to the sagittal plane. It was suggested that an appropriate representation of the MP joint is necessary to better understand the MP joint during locomotion (Smith et al., 2012). However, most previous studies defined the MP joint axis mainly based on anatomical landmarks, e.g., the straight line connecting first and fifth metatarsal heads (Bojsen-Møller and Lamoreux, 1979; Smith et al., 2012). So far, little is known about the realistic orientation and position of the functional rotation axis of the MP joint during locomotion and how it changes with varying locomotor speed (Pohl et al., 2007).

The objective of this study is to investigate the three-dimensional (3D) kinematics of the human MP joint during level walking. The latest three-dimensional motion analysis technique was used to determine the position and orientation of the functional rotation axis of the MP joint in the stance phase of walking for multiple subjects at different speeds. Statistical analysis was conducted to evaluate the difference between the functional MP joint axis and the axis typically defined by the anatomical landmarks in literature. Moreover, the effect of walking speed on MP joint position and orientation was also analyzed statistically. This would provide useful information to improve our understanding of the *in vivo* biomechanical functioning of the human MP joint as well as the propulsive function of the foot during different speeds of locomotion. Furthermore, this may also help in innovating the design of sports and therapeutic footwear, prosthetic lower limbs, and robotic legs, which could be inspired from the nature design of the musculoskeletal system of the human body (Ren et al., 2014).

## Materials and Methods

### Gait measurement

**Figure 1:**
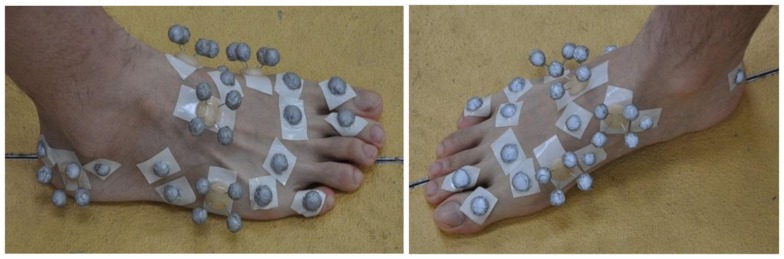
**The infrared marker cluster system used in this study to capture 3D foot motions**. The foot was divided into two segments including tarsometatarsi (hindfoot) and phalanges (forefoot). A set of thermal plastic plates, each carrying four infrared markers, were used to capture the foot segmental motions. A number of hemispherical infrared markers were also attached on the anatomical landmarks.

Six healthy male subjects with normal foot conformation (age: 26.67 ± 2.69 years; weight: 67.17 ± 10.29 kg; height: 175.0 ± 4.43 cm) from a population of postgraduate students, with no previous medical history of foot and lower limb injury, participated in the gait measurement in this study. The subjects provided informed consent in accordance with the policies of local institute ethical advisory committee. All the subjects were instructed to walk barefoot along an indoor walkway at their self-selected slow, normal, and fast walking speeds. A specially designed marker cluster system was mounted firmly on the right feet of the subjects to record the 3D segmental motions of the foot complex (see Figure 1).

A 12 infrared camera motion analysis system (Qualisys, Sweden) was used to capture 3D segmental motions at 150 Hz. Six force plates (Kistler, Switzerland) were used to record the simultaneous ground reaction forces and moments at 1000 Hz. A set of static calibration procedures were undertaken to locate the anatomical landmarks using a calibration wand and reflective markers according to the calibrated anatomical system technique (Cappozzo et al., 1995). The calibration markers were removed before the dynamic walking trials. For each walking speed, the measurement was repeated 15 times to ensure that representative walking data are recorded.

### MP joint definition and parameters

**Figure 2:**
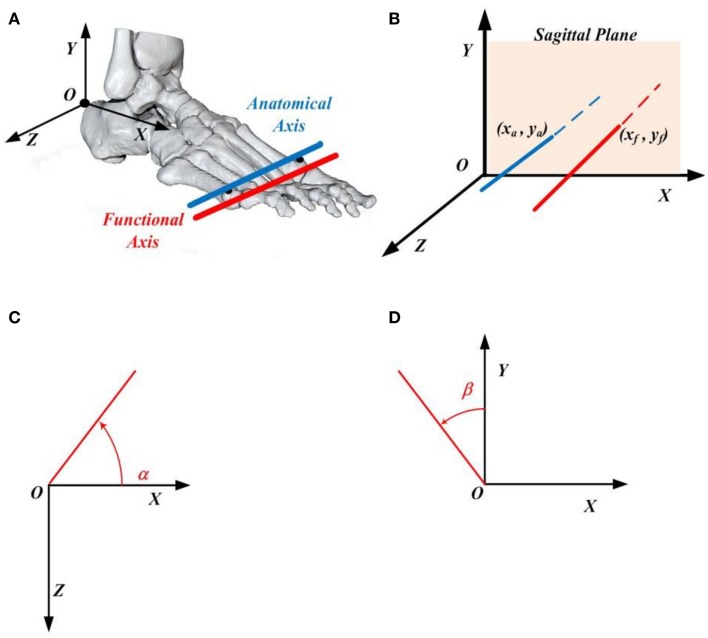
**(A)** The functional axis (red) and anatomical axis (blue) of the metatarsophalangeal joint, where the anatomical axis is defined as the line connecting the first and fifth metatarsal heads. The origin of the foot local coordinate system situates on the upper ridge of the calcaneus bone. **(B)** The position of the functional axis is defined by its intersection point (*x_f_, y_f_*) with the *XOY* plane of the foot coordinate system, whereas the location of the anatomical axis is determined by its intersection point (*x_a_, y_a_*) with the *XOY* plane of the foot coordinate system. **(C)** Angle α made by the functional (or anatomical) axis with respect to the foot *X* axis when projected to the *XOZ* plane of the foot coordinate system. **(D)** Angle β made by the functional (or anatomical) axis with respect to the foot *Y* axis when projected to the *XOY* plane of the foot coordinate system.

Five rigid body segments were defined to represent the lower limb: pelvis, right thigh, right shank, right tarsometatarsi (hindfoot), and right phalanges (forefoot). The 3D anatomical coordinate systems were defined for each individual segment based on the previous studies (Jenkyn and Nicol, 2007; Ren et al., 2008a, 2010). In this study, we have assumed that the five phalanges form a single rigid forefoot segment, whereas the MP joint is considered as a single hinge type joint. The anatomical axis (AA) of the MP joint was defined as the oblique line connecting the first metatarsal head and the fifth metatarsal head (see the blue line in Figure 2A), which is similar to those defined in previous studies (Boonpratatong and Ren, 2010; Graf et al., 2012; Smith et al., 2012). This axis divides the foot into two different segments: hindfoot and forefoot. On the other hand, the functional axis (FA) of the MP joint was defined as the rotational axis between the hindfoot segment and forefoot segment during stance phase of walking (see the red line in Figure 2A). A closed-form algorithm is employed to determine the 3D position and orientation of the FA of the MP joint (Gamage and Lasenby, 2002), which does not require manual adjustment of optimization parameters.

To represent the 3D orientation of the AA and FA of the MP joint, the 3D axes were projected to the *XOZ* and *XOY* planes of the foot local anatomical coordinate system (see Figures 2C,D), where the angles α and β were used to define the axis orientation with counter clockwise being positive based on the right-hand rule (see Figures 2C,D). In addition, to determine the 3D position of the AA and FA of the MP joint, the intersection point (*x_a_, y_a_*) between the AA and the *XOY* plane of the foot local coordinate system, or the intersection point (*x_f_, y_f_*) between the FA and the *XOY* plane of the foot local coordinate system was used (see Figure 2B).

### Data analysis

The raw measured data were processed using GMAS software (Generalized Motion Analysis Software), a MATLAB based software package for 3D kinematic and kinetic analysis of biomechanical multi-body systems (Ren et al., 2005, 2008b). Trials with more than 10 consecutive missing frames were discarded. After the fill-gap processing, the data were filtered using a low pass zero lag fourth-order Butterworth digital filter with a cut-off frequency of 6.0 Hz.

Statistical analyses were conducted to investigate the 3D position and orientation differences between the AA and FA of the MP joint, and also the effect of walking speed on the AA and FA of the MP joint using SPSS 20.0 software (IBM, Armonk, New York, NY, USA). The effects of joint definition (AA or FA) and walking speed on joint position parameters (*x, y*) and orientation parameters (α, β) were analyzed using analysis of variance (ANOVA) with repeated measurements using a linear mixed model approach taking into account intra- and inter-subject variability. The different joint definitions and walking speeds were the fixed effects, and subjects and trials were random effects. Differences between the two joint definitions (AA and FA) and between each pair of walking speeds were tested using Fisher’s least significant difference (LSD) multiple comparison based on the least-squared means, probability by considering *p* < 0.05 as statistically significant.

## Results

**Figure 3:**
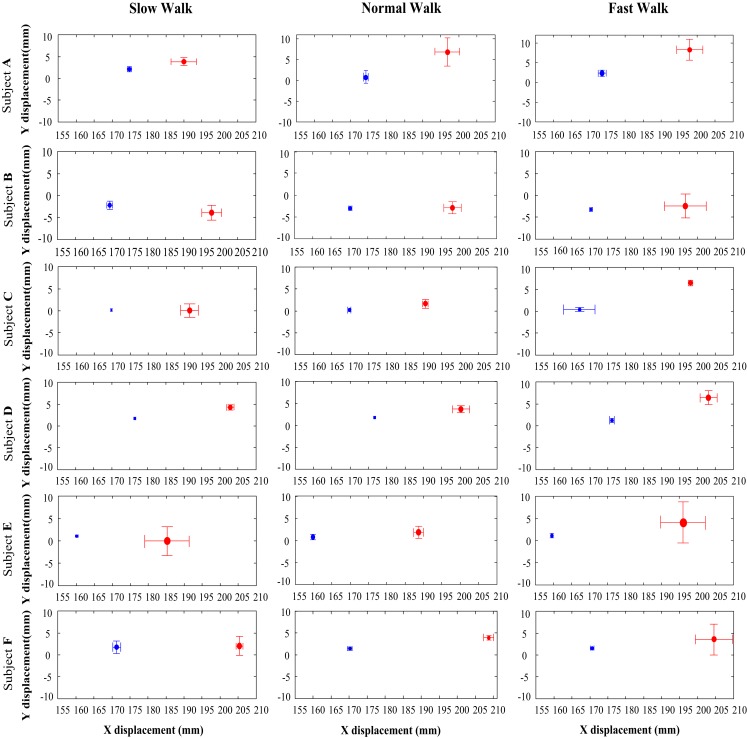
**The *x* and *y* positions of the anatomical axis (blue) and functional axis (red) of the MP joint in the *XOY* plane of the foot coordinate system, for all the six subjects (A to F) across all three walking speeds**. The solid dots indicate the mean *x* and *y* positions, whereas the bars show the 1 SD zones.

The measured data for all the six subjects were processed by using the method described in the preceding section. Figure 3 shows the mean and standard deviation values of the MP joint AA position (*x_a_, y_a_*) and FA position (*x_f_, y_f_*) defined in the *XOY* plane of the foot local coordinate system, where the origin of the coordinate system is at the upper ridge of the calcaneus bone (Ren et al., 2010). The position data of both AA and FA axes for all the six subjects (from subject A to subject F) at all three different walking speeds (slow, normal, and fast) are presented in Figure 3. It can be seen that the FA is consistently anterior to the AA for all subjects across all the three walking speeds with an average position difference of 27.3 mm. In the inferior superior direction (along *Y* axis), both FA and AA positions are close to the origin, and there is no consistent trend in the relative position between the two axes. From the results shown in Figure 3, it appears that the FA of MP joint moves toward a more anterior and superior position with increased walking speed though the displacement in the inferior superior direction (along *Y* axis) is much less than the displacement in the posterior anterior direction (along *X* axis). In contrast to the trend of the FA, it seems that there is no apparent change in the AA position with increased walking speed for all the subjects.

**Figure 4:**
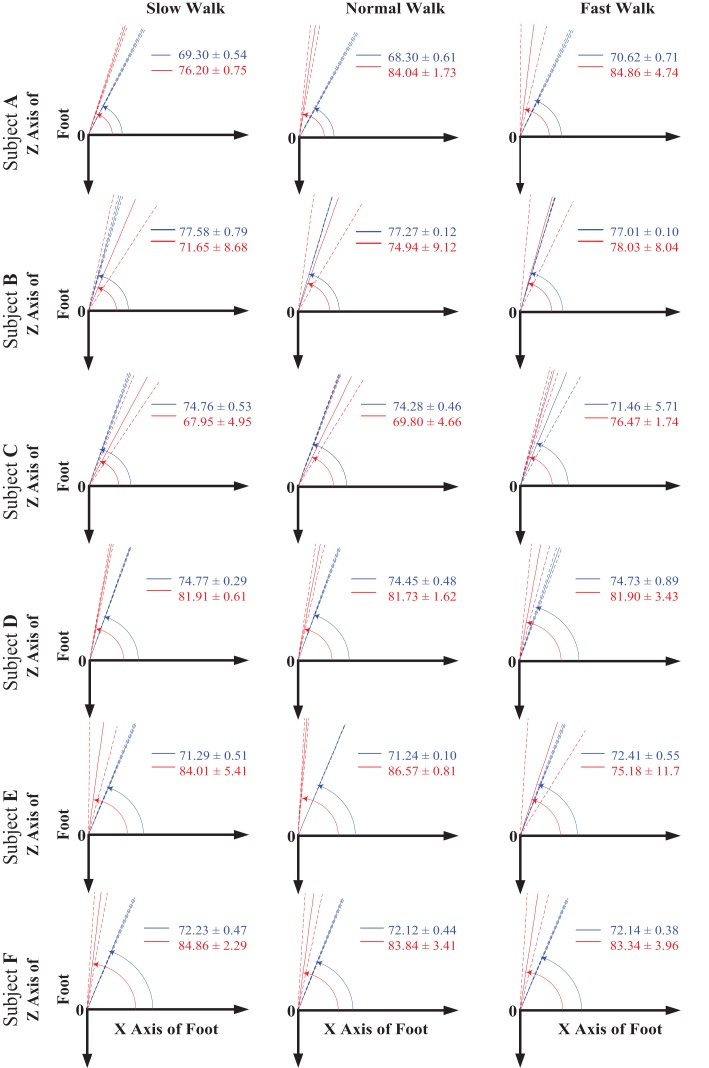
**The orientation angle α made by the functional axis (red) and anatomical axis (blue) with respect to the foot *X* axis when projected to the *XOZ* plane of the foot coordinate system for all the six subjects across all three walking speeds**. The solid lines indicate the mean value of the angle, whereas the dash lines show the 1 SD zones.

Figure 4 shows the mean and the 1 SD zone of the orientation angle α of both AA and FA made with respect to the *X* axis in the *XOZ* plane of the foot coordinate system for all the subjects at all three walking speeds. It can be seen that both AA and FA axes are close to the direction of the foot *Z* axis. According to the right-hand rule, the positive angle α is measured starting from the *X* axis in a counter clockwise direction. From Figure 4, it appears that the FA has an orientation more inclined to the foot *Z* axis than the AA in the transverse plane for all the subjects. Also, it seems that the obliquity of both AA and FA does not change obviously with changing speed.

**Figure 5:**
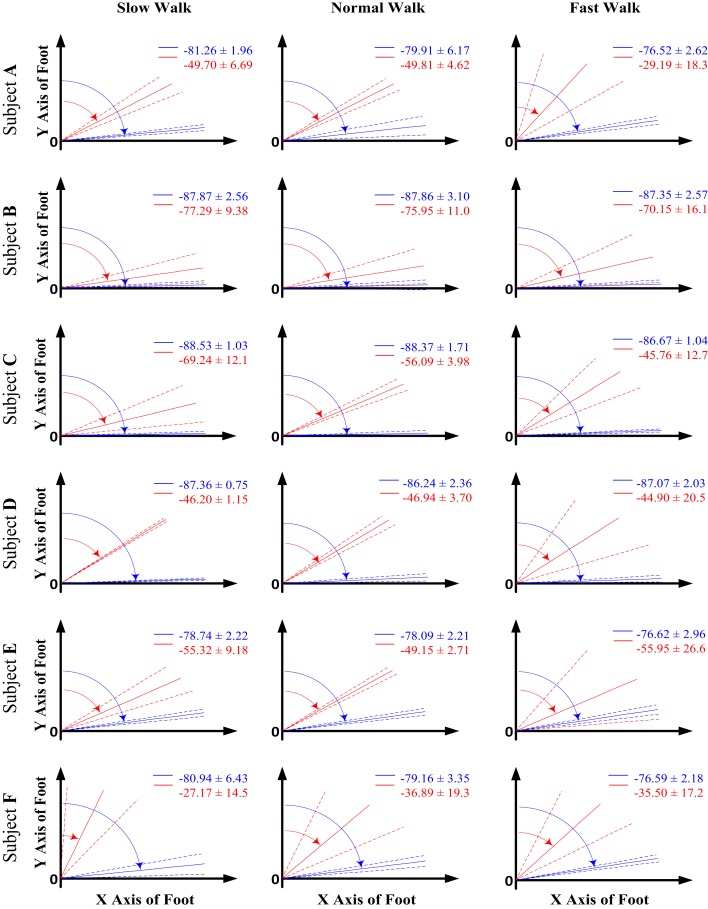
**The orientation angle β made by the functional axis (red) and anatomical axis (blue) with respect to the foot *Y* axis when projected to the *XOY* plane of the foot coordinate system for all the six subjects across all three walking speeds**. The solid lines indicate the mean value of the angle, whereas the dash lines show the 1 SD zones.

Similarly, Figure 5 shows the mean and the 1 SD zone of the orientation angle β of both AA and FA made with respect to the *Y* axis in the *XOY* plane of the foot coordinate system for all the subjects at all three walking speeds. According to the right-hand rule, the negative angle β is measured starting from the *Y* axis in a clockwise direction. It can be seen that the FA shows an apparently different orientation than the AA in the sagittal plane for all the subjects. The FA possesses a higher orientation angle β than the AA leading to a FA direction more inclined to the foot *Y* axis. From the results shown in Figure 5, it seems that the orientation angles in the sagittal plane for both AA and FA do not show particular changes with increased walking speed.

In Table 1, the statistical analysis result on the 3D position and orientation difference between the AA and FA of the MP joint is listed. The position parameters (*x_a_, y_a_*) and (*x_f_, y_f_*) are normalized by the foot length, defined as the distance between the upper ridge of the calcaneus bone and the midpoint between the first and fifth metatarsal heads. It can be seen that statistically significant differences were found for all the 3D orientation and position parameters across all three walking speeds, except for the vertical (in superior inferior direction) position parameter *y* at slow walking speed. The functional rotation axis, FA of the MP joint has a more anterior and superior position than that of the AA, especially during normal and fast walking. This position difference is more significant in superior inferior direction than in anterior posterior direction. The FA of the MP joint shows an anteriorly more medial and more superior orientation than the AA, which is defined as the straight line connecting the first and fifth metatarsal heads. This joint orientation angle difference is more significant in the sagittal plane than in the transverse plane.

**Table 1:** **The result of the statistical analysis of the position and orientation difference between the functional axis (FA) and the anatomical axis (AA) of the MP joint**.

		*x* Normalized by	*y* Normalized by	α (degree)	β (degree)
		foot length	foot length		
Slow walk	FA	1.1496 ± 0.0424^a^	0.0050 ± 0.0193^a^	77.8345 ± 7.9035^a^	-52.6132 ± 24.7011^a^
	AA	0.9989 ± 0.0100^b^	0.0042 ± 0.0096^a^	73.5031 ± 2.7248^b^	-84.2468 ± 4.8971^b^
Normal walk	FA	1.1619 ± 0.0415^a^	0.0153 ± 0.0185^a^	81.2593 ± 6.4280^a^	-51.0727 ± 15.0268^a^
	AA	0.9982 ± 0.0104^b^	0.0029 ± 0.0098^b^	72.8288 ± 2.8084^b^	-82.7051 ± 5.2145^b^
Fast walk	FA	1.1683 ± 0.0390^a^	0.0250 ± 0.0250^a^	80.0469 ± 6.3455^a^	-47.2030 ± 23.4659^a^
	AA	0.9942 ± 0.0187^b^	0.0028 ± 0.0110^b^	73.2755 ± 3.0998^b^	-82.4910 ± 5.6568^b^

*Values are means ± s.e.m. for all trials and all subjects. Identical letters indicate axis groups within a column do not differ significantly from each other (*p* > 0.05)*.

The statistical analysis result investigating the effect of walking speed on the 3D position and orientation of the FA is shown in Table 2. It can be seen that the joint position in the anterior posterior direction shows statistically significant difference when walking speed changes from slow to fast. The FA moves forwards toward a more anterior position with increased speed. More significant change can be seen in the superior inferior direction, the FA moves upwards toward a more superior position appreciably when walking speed increases. No statistically significant effects are found on the FA orientation angles in both the transverse (α) and sagittal (β) planes when walking speed increases from self-selected low to self-selected fast.

**Table 2:** **The result of the statistical analysis of the effect of walking speed on the orientation and position of the functional axis (FA) of the MP joint**.

	*x_f_* Normalized by	*y_f_* Normalized by	α (degree)	β (degree)
	foot length	foot length		
Slow walk	1.1496 ± 0.0424^a^	0.0050 ± 0.0193^a^	77.8345 ± 7.9035^a^	-52.6132 ± 24.7011^a^
Normal walk	1.1619 ± 0.0415^ab^	0.0153 ± 0.0185^b^	81.2593 ± 6.4280^a^	-51.0727 ± 15.0268^a^
Fast walk	1.1683 ± 0.0390^b^	0.0250 ± 0.0250^c^	80.0469 ± 6.3455^a^	-47.2030 ± 23.4659^a^

*Values are means ± s.e.m. for all trials and all subjects. Identical letters indicate speed groups within a column do not differ significantly from each other (*p* > 0.05)*.

Similarly, Table 3 shows the statistical analysis result examining the effect of walking speed on the 3D position and orientation of the AA of the MP joint. No statistically significant change is found in the AA position along the anterior posterior direction. When walking speed increases to normal, the AA position in the superior inferior direction moves apparently toward the inferior direction. There is no statistically significant effect found on the AA orientation angle in the transverse plane (α) with changing speed, whereas a slight increase of the AA orientation angle in the sagittal plane (β) was found when walking speed increases from slow to fast.

**Table 3:** **The result of the statistical analysis of the effect of walking speed on the orientation and position of the anatomical axis (AA) of the MP joint**.

	*x_a_* Normalized by	*y_a_* Normalized by	α (degree)	β (degree)
	foot length	foot length		
Slow walk	0.9989 ± 0.0100^a^	0.0042 ± 0.0096^a^	73.5031 ± 2.7248^a^	-84.2468 ± 4.8971^a^
Normal walk	0.9982 ± 0.0104^a^	0.0029 ± 0.0098^b^	72.8288 ± 2.8084^a^	-82.7051 ± 5.2145^ab^
Fast walk	0.9942 ± 0.0187^a^	0.0028 ± 0.0110^ab^	73.2755 ± 3.0998^a^	-82.4910 ± 5.6568^b^

*Values are means ± s.e.m. for all trials and all subjects. Identical letters indicate speed groups within a column do not differ significantly from each other (*p* > 0.05)*.

## Discussion

The objective of this study is to investigate the 3D orientation and location of the functional joint axis of the MP joint during walking at different speeds. Here, the functional joint axis of the MP joint is defined as the relative rotational axis between the phalanx segments and the hindfoot in the stance phase of walking. In the previous studies, the MP joint was normally defined as a line connecting the first (or second) and fifth metatarsal heads (Boonpratatong and Ren, 2010; Smith et al., 2012), and little is known about the realistic position of the functional joint axis of the MP joint.

Our results show that the 3D orientation and position of the FA of the MP joint is close to the AA defined by the line connecting first and fifth metatarsal heads. However, for the subjects tested in this study, there are some statistically significant differences between the FA and AA. The FA remains anterior to the AA with an average distance about 16% of the foot length across all walking speeds. In the vertical direction, the FA is superior to the AA with an average distance about 2% of the foot length during normal and fast walking, whereas, the FA shows a higher obliquity than AA with an anteriorly more medial and superior orientation. This suggests that using the AA to represent the MP joint may result in overestimated MP joint moment and power and also underestimated muscle moment arms for MP extensor muscles.

It was found that walking speed has statistically significant effect on the position of the FA though the FA orientation remains unchanged with varying speed. When walking speed increases, the FA moves forwards and upwards toward a more anterior and more superior position. This joint axis shift toward the ground reaction force vector in the late stance of walking will result in decreased moment arm of ground reaction force and also simultaneously increased moment arm of MP extensor muscles, and hence will increase the EMA of MP extensor muscles (Biewener, 1989, 1990, 2003; Biewener et al., 2004; Winter, 2005). In addition, a forward shift of the MP joint axis will result in an increased lever distance to the ankle joint, and may moderate the angular velocity increase with increasing walking speed. This may help to maintain the contraction velocity of the MP extensor muscles in the power optimal region (Biewener, 2003). It appears that a variable gear mechanism also exists in the MP joint especially in the late stance of walking (Carrier et al., 1994).

This study has some limitations. A single hinge type joint was assumed to represent the relative motion between the phalanges and the rearfoot throughout the stance phase of walking. As suggested by previous studies, two different axes may exist at the MP level during locomotion (Bojsen-Møller, 1978; Bojsen-Møller and Lamoreux, 1979). Our future works will involve the investigation of the 3D positions and transition of multiple MP axes. In addition, two rigid bodies (phalanges and hindfoot) were assumed to represent the motion of the foot complex by using multiple marker clusters in the gait measurement. However, in reality, some appreciable relative motions may occur between phalanges or the hindfoot bones. Thus, more complicated models may be needed in the future to better understand the 3D kinematics at MP joint. Furthermore, bone-pin markers rather than skin mounted markers may help to reduce the skin artifact involved in the gait measurement (Nester et al., 2007).

## Conclusion

This study reveals many factors at the distal end of the foot, which may contribute to the locomotor function of the human foot complex. This includes not only the obliquely oriented functional rotation axis but also the relative position of the axis with respect to the hindfoot bones. The position of the FA of the MP joint is found to be anterior and superior to the AA with higher obliquity. Furthermore, with increasing speed the FA shifts more anterior. This forward and upward shift of the FA with increasing walking speed may help to moderate the muscular effort, maximize the locomotor efficiency, and reduce the risk of injury. This study may help us to better understand the contribution of the intrinsic foot structure to the propulsive function of the foot during locomotion at different speeds. Furthermore, this may also help in improving the design of sports and therapeutic footwears, prosthetic lower limbs, and robotic legs.

## Conflict of Interest Statement

The authors declare that the research was conducted in the absence of any commercial or financial relationships that could be construed as a potential conflict of interest.
